# Ebola Virus Nucleocapsid-Like Structures Utilize Arp2/3 Signaling for Intracellular Long-Distance Transport

**DOI:** 10.3390/cells9071728

**Published:** 2020-07-19

**Authors:** Katharina Grikscheit, Olga Dolnik, Yuki Takamatsu, Ana Raquel Pereira, Stephan Becker

**Affiliations:** 1 Institute of Virology, Philipps University Marburg, Hans-Meerwein-Str. 2, 35043 Marburg, Germany; katharina.grikscheit@posteo.de (K.G.); dolnik@staff.uni-marburg.de (O.D.); yukiti@niid.go.jp (Y.T.); 2 German Center for Infection Research (DZIF), Partner Site: Giessen-Marburg-Langen, Hans-Meerwein-Str. 2, 35043 Marburg, Germany; 3 Department of Virology I, National Institute of Infectious Diseases, Tokyo 208-0011, Japan; 4 Oxford Nano Imager, Linacre House, Banbury Rd, Oxford OX2 8TA, UK; pereira.arr@gmail.com

**Keywords:** Ebola virus, actin cytoskeleton, nucleocapsid transport, Arp2/3 complex

## Abstract

The intracellular transport of nucleocapsids of the highly pathogenic Marburg, as well as Ebola virus (MARV, EBOV), represents a critical step during the viral life cycle. Intriguingly, a population of these nucleocapsids is distributed over long distances in a directed and polar fashion. Recently, it has been demonstrated that the intracellular transport of filoviral nucleocapsids depends on actin polymerization. While it was shown that EBOV requires Arp2/3-dependent actin dynamics, the details of how the virus exploits host actin signaling during intracellular transport are largely unknown. Here, we apply a minimalistic transfection system to follow the nucleocapsid-like structures (NCLS) in living cells, which can be used to robustly quantify NCLS transport in live cell imaging experiments. Furthermore, in cells co-expressing LifeAct, a marker for actin dynamics, NCLS transport is accompanied by pulsative actin tails appearing on the rear end of NCLS. These actin tails can also be preserved in fixed cells, and can be visualized via high resolution imaging using STORM in transfected, as well as EBOV infected, cells. The application of inhibitory drugs and siRNA depletion against actin regulators indicated that EBOV NCLS utilize the canonical Arp2/3-Wave1-Rac1 pathway for long-distance transport in cells. These findings highlight the relevance of the regulation of actin polymerization during directed EBOV nucleocapsid transport in human cells.

## 1. Introduction

The dynamic actin cytoskeleton is commonly utilized by pathogens for entry, exit and their intracellular assembly [1]. While for some intracellular bacteria and DNA viruses, the mechanism of hijacking the actin cytoskeleton is described in detail, the remodeling of actin through highly pathogenic RNA viruses such as filoviruses remains poorly understood [1,2]. Recently, it has been demonstrated that the intracytoplasmic transport of the highly pathogenic Marburg virus (MARV) and the Ebola virus (EBOV) depends on actin polymerization, but the detailed mechanisms and cellular interaction partners remain largely unknown [3,4,5].

MARV and EBOV belong to the family of *Filoviridae*, which are filamentous, enveloped viruses containing a single strand, negative-sense RNA genome, and which cause severe fevers in humans with very high fatality rates [6]. Strikingly, filoviral RNA encodes for only seven structural proteins, which are multifunctional, and diversely hijack, disable or reorganize cellular pathways [7]. For example, EBOV VP24 and VP35 are able to block the cell’s interferon system, resulting in the viruses’ escape from the innate immune response [8].

A hallmark of the filoviral life cycle is the formation of perinuclear inclusion bodies, where transcription and replication occur and de novo viral nucleocapsids are formed [9]. These nucleocapsids are mainly composed of the nucleoprotein (NP) that encapsidates the viral RNA in highly organized helical structures (approximately 1000 nm long and 50 nm in diameter) [10]. Furthermore, nucleocapsids contain the NP-binding proteins VP24 and VP35, as well as polymerase L and the transcription initiation factor VP30 [10,11,12].

Following their assembly in the inclusion bodies, individual nucleocapsids have to be transported over long distances through the cytoplasm to reach the cell periphery [3,5,9]. It has been shown that they accumulate in filopodia structures. Finally, nucleocapsids acquire their envelope at the plasma membrane, containing the viral trans-membrane glycoprotein (GP) and the viral matrix protein VP40, triggering the budding of the filamentous infectious virus particles. [8]. The directed transport is required for the rapid distancing away from the inclusion bodies, and to transfer functional nucleocapsids to the plasma membrane. It has been shown that individual single mutations within the nucleocapsid proteins critically affect long-distance transport, thereby leading to a significant delay and reduction of the release of filamentous nucleocapsids [13,14,15].

**Figure 1:**
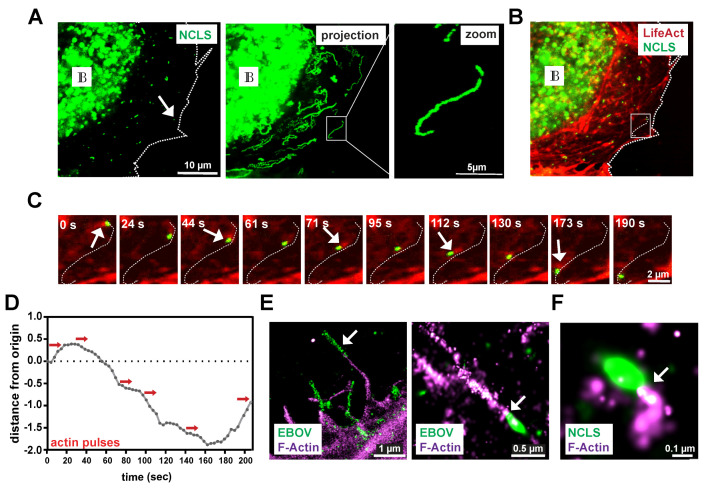
Ebola virus (EBOV) nucleocapsid-like structures induce polar actin tails during transport. (**A**) Exemplary still image from a movie of a Huh7 cell expressing NP, VP24, VP35 and VP30-GFP (left panel). During imaging, NCLS are tracked for 2 min producing long-distance trajectories as depicted in the maximum intensity projection of the movie (right panel). The white arrow highlights an individual track. IB labels inclusion bodies. (**B**) Co-imaging of actin using LifeAct-CLIP (stained with Alexa-657 dye) reveals a dense and dynamic actin network. White inset is magnified in (**C**) showing the time course of an individual NCLS (white arrow, Figure 1**B**), revealing a pulsative actin tail located on one site of the subviral particle during movement (see also Movie S1). (**D**) Graph showing the movement of this NCLS over time. The red arrows indicate actin pulses observed. (**E**,**F**) STORM images showing NCLS or EBOV nucleocapsids immunolabelled with NP (green) and stained with Phalloidin (magenta). (**D**) The left panel shows a filamentous virus particle likely prior to budding (white arrow). Individual intracellular nucleocapsids reveal a preserved actin tail (white arrow). (**E**) Huh7 cells were transfected with NP, VP24 and VP35, and fixed after 24 h. The samples were stained with anti-NP and Phalloidin, and prepared for STORM microscopy. The zoomed image shows an individual NCLS with a preserved actin tail (white arrow).

**Figure 2:**
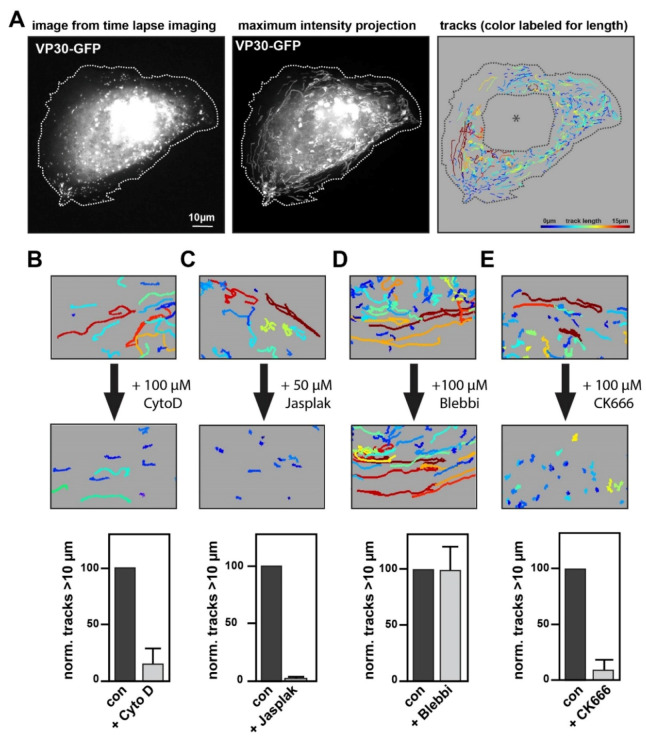
Analysis of NCLS transport using actin-modulating drugs. (**A**) Exemplary still image from a movie of a Huh7 cell expressing NP, VP24, VP35 and VP30-GFP (left panel). For live cell imaging, NCLS are tracked through the cell over 2 min with images captured every 900 ms. While the middle panel shows the maximum-intensity projection of this movie, the right panel depicts the same movie quantified using the spot algorithm (Imaris). The tracks are color-coded for length. To exclude artefacts, we semi-manually deleted areas with strong accumulation of VP30-GFP, such as the inclusion bodies (asterisk). (**B**–**E**) Huh7 cells transfected with NCLS (VP30-GFP) were recorded for 2 min. This was followed by a short incubation with cytoskeletal-modulating drugs after which the cells were re-recorded. Per experiment three cells were recorded and all tracks > 20 s were quantified. The pictures show a magnified area after quantification with Imaris. Cells were incubated with (**B**) 100 µM Cytochalasin D, (**C**) 50 µM Jasplakinolide, (**D**) 100 µM para-nitro-Blebbistatin or (**E**) 100 µM CK666 following the first imaging. The graphs depict the normalized number of tracks >10 µm, error bars show the SD of *n* = 3 experiments.

While other viruses utilize the microtubule network for intracellular distribution, [16] the transport of nucleocapsids of MARV as well as EBOV is entirely blocked by the pharmacological inhibition of actin polymerization [3,4]. However, the role of actin nucleators and the regulation of their upstream effectors in this process remains elusive. One major obstacle in studying filoviruses is the requirement of high containment laboratories, restricting cell biological experiments that could accelerate our understanding of the critical steps in the virus life cycle. To overcome this problem, we recently established a novel transfection-based system that only requires three viral proteins to produce nucleocapsid-like structures (NCLS), which highly resemble EBOV nucleocapsids in their structure as well as their intracellular dynamics [5]. Through the co-expression of GFP-tagged VP30, which faithfully labels NCLS, it is possible to monitor intracellular transport mechanisms with real time imaging under normal laboratory conditions (biosafety level 1) conditions (Figure 1A,B and Figure 2A).

The host cell contains a highly dynamic actin cytoskeleton that is required for many essential cellular processes, such as cytokinesis, contractility and motility [17,18]. Filamentous actin (called F-actin) is assembled from monomeric globular G-actin, and this polymerization, as well as depolymerization, is highly regulated through a plethora of different cellular factors [19,20,21]. With this high power of regulation, the cell is able to assemble and coordinate diverse structures, such as the strong cortical actin network stabilizing the cell cortex, short and highly dynamic filaments that are involved in vesicular trafficking, and filament networks that form membrane protrusions such as the filopodia and lamellipodia required for cell motility or cell–cell contact formation [22,23,24,25]. The Arp2/3 (actin-related protein) complex efficiently nucleates the actin filaments typically attached to mother filaments to form the highly branched networks that (amongst other things) enable lamellipodia to rapidly adapt during cell migration [26]. This active protein complex has to be tightly regulated, and requires activation for polymerization [27]. So-called nucleation promoting factors (NFPs), such as WAVE1 and WASP proteins, directly induce Arp2/3 complex activity [27,28], and are themselves activated downstream of RhoGTPase signaling in a highly spatial–temporal manner [29].

The Arp2/3 complex is highly conserved, and different pathogens, including *Listeria monocytogenes* and vaccinia virus, utilize its activity for viral intracellular transport steps [30]. For example, the membrane-integrated *Listeria* protein ActA mimics the NPF WASP, thereby recruiting and activating the Arp2/3 complex [31,32,33]. The Arp2/3 complex in turn induces local actin polymerization, resulting in so-called actin comet tails that efficiently propel the bacterium through the cytoplasm, pushing it into neighboring cells. Furthermore, actin comet tails have been previously observed at EBOV nucleocapsids [3]; however, the mechanism by which viral nucleocapsids use actin dynamics for their transport has not been described in detail.

In this study, we employ our recently established live cell imaging approach to delineate the cellular pathways by which EBOV exploits host actin signaling, and extend the previously applied manual quantification approach to a semi-automatic high throughput method. Using small inhibitory compounds and siRNA-mediated knockdown, we demonstrate that Arp2/3 complex activity downstream of Rac1 is critically involved in the directed long-distance transport of EBOV nucleocapsid structures. Furthermore, through co-visualization of NCLS transport with the actin marker LifeAct, we detected pulsative actin tails accompanying the movement of NCLS through the cytoplasm, which requires Arp2/3 activity.

## 2. Materials and Methods

### 2.1. Cells and Viruses

Huh7 (human hepatoma) cells were cultured in DMEM (Life Technologies, Carlsbad, CA, USA) supplemented with 10% (*v*/*v*) fetal calf serum (FCS) (PAN Biotech), 5 mM l-glutamine, 50 U/mL penicillin and 50 μg/mL streptomycin (Life Technologies) and grown at 37 °C with 5% CO_2_. For live cell imaging experiments, cells were kept in phenol-free Leibovitz’s medium (Life Technologies) with PS/Q, non-essential amino acid solution and 20% (*v*/*v*) FCS.

The virus used in this study was based on EBOV Zaire (Strain Mayinga; GenBank accession no AF27200 (National Center for Biotechnology Information, Bethesda, MD USA)). The experiments with infectious EBOV virus were performed in the BSL-4 facility at the University of Marburg.

### 2.2. Transfections, Plasmids, siRNA and Inhibitors

The plasmids coding for EBOV proteins (pCAGGS-NP, -VP35, -VP24) and pCAGGS-VP30-GFP were described previously [34]. Transient transfections of Huh7 cells were carried out using TransIT-LT1 (Mirus, Madison, WI, USA) according to the manufacturers’ instructions with 3 µL reagent per 1 µg plasmid DNA [5]. Transfection of siRNAs was performed using DharmaFECT (Horizon, Waterbeach, UK) using 25 nM siRNA and 5 µL transfection reagent in Opti-MEM (Thermo Fisher, Waltham, MA, USA). Huh7 cells were transfected with siRNA in a 6-well µ-slide, seeded to a 4-well µ-slide (IBIDI) and then transfected with the plasmids encoding for the viral proteins (200 ng/µL VP30-GFP, 30 ng/µL VP24, 200 ng/µL VP35 and 200 ng/µL NP). Then, NCLS movement was monitored 24 h later through detection of VP30-GFP. The FlexiTube siRNA used were purchased from Qiagen and diluted to 10 µM. The siRNAs used in the study were HS-ACTR3_5 (AAAGTGGGTGATCAAGCTCAA), HS_RAC1_6 (ATGCATTTCCTGGAGAATATA), HS_WASF1_3 (CAAGAACGTGTGGACCGTTTA) and HS-CDC42_16 (5′-CATCAGATTTTGAAAATATTTAA 3′). For treatment with inhibitors, individual transfected cells (NP, VP24, VP35 and VP30-GFP) were monitored, then the inhibitor was added to the cells in the appropriate dilution and after a short incubation the very same cell was imaged. Cytochalasin D, Jasplakinolide and NSC 23766 were obtained from Sigma Aldrich, and CK666 was purchased from EMD Millipore.

### 2.3. Antibodies and Reagents for Microscopy

The following primary antibodies were used in this study: polyclonal anti-chicken EBOV NP [35], polyclonal rabbit anti-Wave1 (Sigma Aldrich, St. Louis, MO, USA), mouse anti-Arp3 (Sigma, A5979), mouse anti-Rac1 (Cytoskeleton) and mouse anti-Tubulin (Sigma). The corresponding secondary antibodies were anti-mouse-HRP and anti-rabbit-HRP (DAKO, Jena, Germany), and anti-chicken-Alexa555 (Invitrogen, Carlsbad, CA, USA). Western Blot analyses were performed as described previously [5]. SNAP/CLIP technology was used to visualize actin by using LifeAct-CLIP. LifeAct-CLIP was cloned using standard cloning procedures and then transfected with the NCLS system. For co-visualization of actin and NCLS transport, transfected cells cells were incubated with the dye CLIP-647 (1:500 diluted in media, NEB) for 30 min prior to the experiment, then washed and replaced with Leibovitz medium for live cell imaging.

### 2.4. Confocal Microscopy, Live Cell Imaging and STORM Microscopy

Huh7 cells were fixed using 4% PFA/DMEM, permeabilized with 0.3%Triton-X100/PBS and blocked with blocking buffer containing 3% glycerol, 2% BSA, 0.2% Tween and 0.05% NaN_3_. Primary antibodies as well as secondary antibodies were diluted in blocking buffer and both were incubated for 1 h at RT. The actin cytoskeleton was visualized with Phalloidin conjugated with Alexa647 (Cytoskeleton). For STORM analysis of transfected cells, cells were fixed with 4% PFA/PBS, permeabilized with 0.3% Triton-X100 and then stained with Phalloidin-Alexa647 (1:50) in PBS for 48 h at 4 °C, before being immunolabelled for NP using anti-chicken NP (1:1000) in blocking buffer followed by an incubation with anti-chicken-Alexa555 (Invitrogen, 1:250). Every instance of Phalloidin or antibody staining was followed by a post-fixation step using 4% PFA/PBS. Cells infected with EBOV were fixed with 4% PFA/PBS and permeabilized, then stained with Phalloidin for 1 h in the BSL4 lab, followed by 48 h incubation with 4% PFA/DMEM. Cells were then blocked and immunolabeled as described above and re-stained with Phalloidin for 48 h. For STORM analysis, the coverslip was mounted in switching buffer (1 M MEA-HCl in Glucose buffer containing catalase, TCEP and glucose oxidase).

Dual color live-cell imaging was performed on a SP8 confocal laser scanning microscope (Leica) equipped with a 64 × 1.4 oil objective, and movies for single color live cell imaging were recorded using a Nikon ECLIPSE TE2000-E microscope with a 64x-oil objective. The movies for quantification were acquired every 900 ms for 120 s. All live cell imaging was performed at 37 °C. STORM images were acquired using the Oxford Nanoimaging system (ONI). The light was collected by the 100× objective and imaged onto the EM-CCD camera at 30 ms per frame. Images were processed using the NimOS localization software (Oxford Nanoimaging).

### 2.5. Particle Tracking, Quantification and Statistics

Image processing was performed with Imaris (Bitplane, Oxford Instruments, Abingdon, UK), FIJI (NIH) and Photoshop CS6 (Adobe, San Jose, CA, USA). For figure design, we used Adobe Illustrator. NCLS tracking was performed using Imaris (Bitplane). To this end, VP30-GFP signal was monitored every 900 ms over 2 min and then moving NCLS were analyzed using the “spots feature”, a presetting that has been optimized for cell and particle tracking within the software. Within the “spots” feature, we determined an algorithm that automatically classifies objects of over 0.4-µm diameter appearing continuously for at least 20 s as NCLS. The positions of the structures were then calculated in each image of the 2-min movie. From these data, the software is able to calculate trajectories using an autoregressive motion algorithm. After the computational calculation and visualization of all tracks, we semi-manually filtered out particle aggregations, as well as GFP signals derived from inclusion bodies, by deleting spots within certain areas within the cell (see Figure 2A), resulting in approximately 400–800 tracks per cell. We filtered the data (e.g., for tracks >10 µm or straightness > 0.7) which were then used for quantification. This algorithm was then applied to all files of a set of experiments. Each experiment was performed in at least three independent repetitions. Statistical analyses were performed with Prism x (GraphPad, San Diego, CA, USA).

## 3. Results

### 3.1. Nucleocapsid Movement Is Accompanied by Pulsative Actin Tails

Here, we employ a novel live cell imaging system to monitor the long-distance movement of EBOV NCLS, in which only three viral proteins are expressed, namely, NP, VP24 and VP35 (Figure 1A, left panel) [5]. Using VP30-GFP, we monitor the intracellular transport of NCLS for two minutes, and the corresponding maximum-intensity projection reveals that a robust number of NCLS (6.9% ± 2.7%) are transported in a highly directed fashion over long distances (>10µm–20µm), now referred to as long-distance trajectories (Figure 1A, right panel). However, due to defective assembly or saturation of the cellular transport machinery, we also detect many VP30-GFP-positive NCLS that do not move, or travel only short distances (Figure S1A).

**Figure 3:**
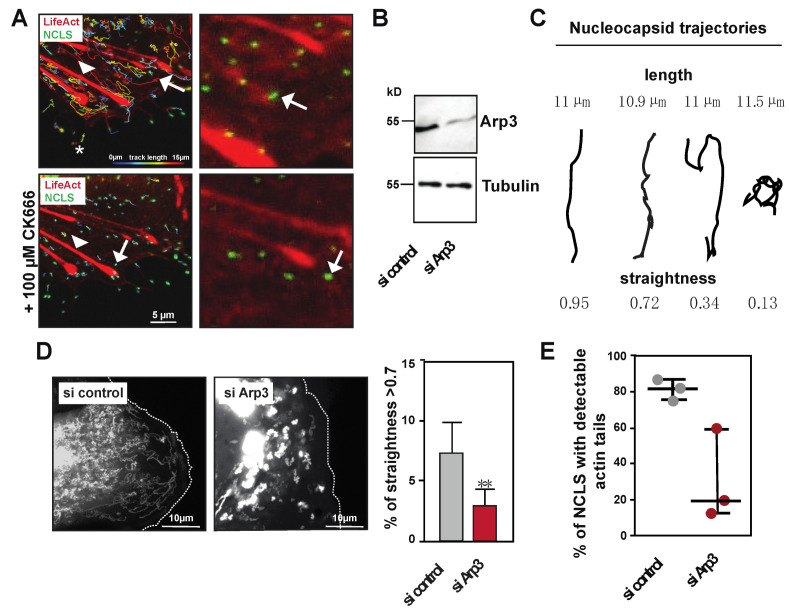
NCLS induction of actin tails depends on Arp2/3 activity. (**A**) Huh7 cells transfected with NP, VP24 and VP35 and LifeAct-CLIP were monitored over time. The left panels show an overlay image of the tracks as calculated by Imaris (color coded for length) and LifeAct-CLIP (still image from movie). Note, CK666 incubation does not interfere with filopodia (asterisk) or stress fibers (arrow head) (see Movie S2). The zoomed image (right panels) show still images of the movie, revealing that CK666 treatment abolishes actin tail formation (white arrows). (**B**) Western Blot showing the depletion of Arp3 after siRNA treatment. (**C**) Cartoon showing different movements of trajectories and their corresponding straightness value. (**D**) Left panel shows maximum intensity projections of time lapse imaging of Huh7 cells transfected either with control or Arp3 siRNA. The right panel shows the graphical analysis of live cell imaging comparing all tracks > 10 µm in cells transfected either with control or Arp3 siRNA. Note, the relative amount of tracks with a straightness > 0.7, representing directed long-distance transport, is reduced in cells transfected with Arp3 siRNA (taken from *n* = 3 experiments, * *p* = 0.0027, Mann–Whitney Test). (**E**) Graph shows the percentage of trajectories with pulsative actin tails in live cell imaging (*n* = 3, error bars indicate mean with standard deviation).

Actin tail formation at EBOV nucleocapsids was detected in an earlier study [3]. Therefore, we aimed here to determine whether or not the established transfection system can recapitulate the detection of actin tails at NCLS as well. In cells that co-express the actin marker LifeAct to visualize the actin cytoskeleton (Figure 1B), we observed that NCLS induces actin tails during their movement through the cell (movie S1), thereby further highlighting the relevance of this transfection system to the study of the transport of EBOV nucleocapsids. Here, we further assessed the movement of an individual NCLS-track over time, starting from its origin and following it through the cell (inset of Figure 1B is magnified in Figure 1C, white arrows). In Figure 1D, the red arrows indicate actin pulses during movement, which appear to correlate with the altered movement of NCLS, such as changes in direction (Figure 1C, Movie S1). While with the live cell imaging setting we used approximately 80% of the tracks that moved in a directed fashion revealed detectable actin tails at their rear end (Figure 3E, control siRNA), we cannot exclude the possibility that smaller NCLS induce actin polymerization below the detection limit, due to the high intracellular background level of LifeAct.

While live cell imaging was applied to capture rapid and transient phenomena, the image resolution remained low. To precisely determine the localization of actin tails at filoviral nucleocapsids, we decided to use a high resolution, single molecule detection technique (STORM). To this end, we either transfected cells with NP, VP35 and VP24, or infected cells with EBOV and fixed them after 24 h. This was followed by the staining of the actin cytoskeleton and immunolabelling for the viral NP protein. Intracellular NCLS or EBOV nucleocapsids appear as elongated NP-positive structures (Figure 1E,F). However, compared to NCLS in transfected cells, filamentous nucleocapsids in cells infected with EBOV were commonly detected along the plasma membrane and within filopodia, likely representing virus particles prior to or during budding, thereby supporting previous observations from live-cell imaging and electron microscopy (Figure 1D, left panel) [3]. Co-staining with Phalloidin revealed actin tail structures in the vicinity of the nucleocapsids in infected cells and transfected cells (Figure 1D,E), which were less frequent in infected cells. This finding is likely a consequence of the very high spatial-temporal dynamics of actin tail formation, which do not allow a quantifiable detection in fixed cells. Further, in the minimalistic transfection system, NCLS remain in the cytosol and are not released, thus the detection of NCLS with actin tails may occur more frequently. Additionally, it cannot be excluded that in infected cells, the long fixation procedure required for the handling of BSL4 samples might interfere with the fixation of these transient actin structures.

### 3.2. Characterization of NCLS Transport Dynamics

To measure and characterize the long-distance movement of NCLS in a semi-automated manner, we analyzed our time-lapse movies with a tracking tool within the software Imaris. To this end, we set up an algorithm to follow NCLS, and filtered for trajectories > 10 µm that show mean velocities comparable to those measured in previous reports (here, 167 ± 58 nm/s compared to 187 ± 67 nm/s as in Takamatsu et al., 2018 [5]) (Figure 2A).

These studies demonstrated that the long-distance movement of EBOV nucleocapsids, as well as NCLS, is sensitive to the inhibition of actin polymerization [3,5]. Here, we recapitulated these experiments by monitoring NCLS movement followed by an incubation with an actin-modulating drug, and then re-recorded the very same cell. Using this system, we ensured that the imaged cells were capable of showing long-distance trajectories, and would consequently enable us to analyze alterations in NCLS movement. As observed previously, the application of the inhibitor of actin polymerization, Cytochalasin D, results in a rapid and strong decrease in any long-distance trajectories of NCLS, quantified as a highly reduced number of tracks over 10 µm (Figure 2B) [3,5]. Expanding on previous results, we further incubated the cells with the actin polymerization and stabilization agent Jasplakinolide, resulting in a similar phenotype, thereby indicating that indeed not only actin polymerization, but also depolymerization, is required for the long-distance transport of NCLS (Figure 2C). Treatment with Blebbistatin (here we used para-nitro-Blebbistatin to avoid phototoxicity in live cell experiments) did not interfere with long-distance movement, suggesting that myosin II activity is not required for the long-distance transport of NCLS (Figure 2D). Taken together, these findings reinforce the importance of actin regulation and dynamics in the long-distance transport of NCLS.

### 3.3. Arp2/3 Complex Activity Is Required for Actin Tail Formation and Directed Long-Distance Transport

Previous experiments showed that inhibitors of Arp2/3 affected the long-distance movement of EBOV nucleocapsids [3]. To confirm and further elaborate on these results, we used the minimalistic transfection system to analyze how treatment with an Arp2/3 inhibitor (CK666) affects NCLS movement [36]. Movie S2 shows that blocking Arp2/3 activity rapidly abolishes any directed long-distance movement of NCLS, and that minimal movement likely derives from Brownian motion (Figure 2E, Movie S2). When dissecting different pools of tracks, we observed that treatment with CK666 strongly decreases tracks >10 µm, subsequently resulting in increases in tracks < 5 µm, which also show altered mean speeds (Figure S1A,B). In cells treated with CK666, we observed a slight reduction in the overall NCLS (−19 ± 7%), which might have resulted from the decreased NCLS transport from the inclusion bodies into the cytoplasm (Figure S1C). Furthermore, Movie S2 reveals that the abolishment of NCLS transport, using CK666, coincides with the loss of actin tail formation (Figure 3A, tracks are color-coded for length and actin is visualized with stills from the confocal movie). Time-lapse imaging further demonstrates that Arp2/3-independent structures, such as filopodia or stress fibers, are not compromised during the observed time frame, thereby highlighting a specific role for Arp2/3-dependent actin polymerization in the directed long-distance transport of NCLS (Movie S2).

Next, we continued with the depletion of one protein within the Arp2/3 complex, namely Arp3, using siRNA transfection to further evaluate its role in actin tail-dependent NCLS transport (Figure 3B). The siRNA depletion of Arp3 likely results in the destabilization or reduction of functional Arp2/3 complexes, and does not entirely block all long-distance movement of NCLS, as observed under treatment with CK666. Thus, we assessed whether the remaining tracks showed altered directionality in cells depleted for Arp3, through the calculation of trajectory straightness (ratio of distance to length) (Figure 3C). We determined that tracks with a straightness > 0.7 (Figure 3C), and indeed the depletion of Arp3, interferes with the total number of straight tracks, further highlighting a role for Arp2/3 activity in the long-distance transport of NCLS (Figure 3D,E). Next, we compared the formation of actin tails in control and Arp3-depleted cells. While approximately 80% of the NCLS that travel over long distances showed pulsative actin tails, cells depleted for Arp3 showed a decreased level of detectable actin polymerization at their rear end (Figure 3E,F). Taken together, these results indicate that Arp2/3 activity is required for the efficient directed long-distance transport of NCLS, and that the Arp2/3 complex is directly involved in the formation of actin tails in Huh7 cells.

### 3.4. Identification of Rac1/WAVE1/Arp2/3 Signaling Network that Is Involved in Long-Distance Transport of NCLS

**Figure 4:**
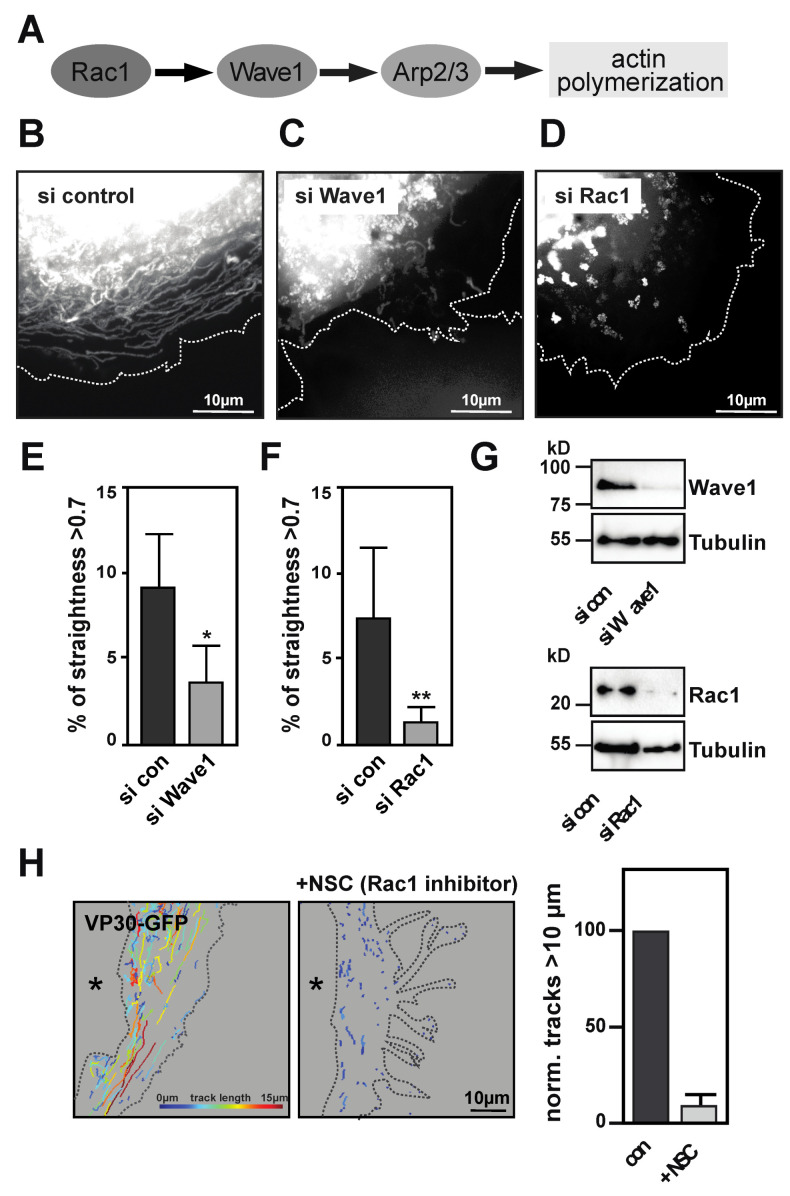
Identification of a Rac1/Wave1/Arp2/3 signaling. (**A**) Cartoon depicting canonical Arp2/3 signaling downstream of Rac1 [39]. (**B**–**D**) Images show maximum intensity projection of time-lapse images of cells recorded for 2 min; images were captured every 900 ms. Cells were transfected with control siRNA (**B**), siRNA against (**C**) Wave1 (*WASF1*) or (**D**) Rac1. (**E,F**) Analysis of live cell imaging comparing relative straightness of tracks > 10 µm and a straightness > 0.7 after either Wave1 siRNA (**E**) or Rac1 siRNA (**F**) transfection. Cells were transfected with siRNA against (**B**) Wave1 (*WASF1*) or (**C**) Rac1. Note that siRNA treatment against Wave1 and Rac1 results in a decrease in straight long-distance trajectories (taken from three independent experiments, at least 5 cells per experiments, ns = non-significant, ** *p* > 0.001, * *p* > 0.01). (**G**) Western Blots showing effective siRNA depletion of Wave1 and Rac1. (**H**) Incubation of Huh7 cells transfected with NCLS (VP30-GFP) was first recorded for 2 min, then they were incubated with 100 nM Rac1 inhibitor (NSC23766) for 1 h and then reimaged. The overview images reveal that NCS23766 also inhibits long-distance transport. Tracks are color-coded for mean speed (6 cells in *n* = 3, *p* < 0.0079, *t*-test).

The Arp2/3 complex resides, in inactive status, within the cell, and requires activation by NFPs, such as N-WASP or Wave proteins, that bind and activate the Arp2/3 complex via their conserved VCA-domain (Figure 4A) [29]. As it was recently demonstrated that the inhibition of *N*-WASP using Wiskostatin did not interfere with the long-distance transport of EBOV nucleocapsids [3], we focused on Wave1, an alternative activator of the Arp2/3 complex. Interestingly, siRNA directed against WASF1 (Wave1) faithfully recapitulated the phenotype of Arp3 siRNA treatment, leading to the reduced straightness of the NCLS (Figure 4B). This indicates that activation of the Arp2/3 complex downstream of Wave1 is involved in the directionality of long-distance trajectories. Wave1 activity itself can be controlled by the RhoGTPase Rac1 (Figure 4A–C) [37,38]. Again, the treatment of Huh7 cells with siRNA against Rac1 resulted in a decreased level of directed long-distance trajectories (Figure 4D,F,G). In contrast, the depletion of Cdc42, another member of the RhoGTPase family, did not affect the straightness of trajectories (Figure S1D), thereby further reinforcing the inference that Rac1 activity is involved in NCLS transport (Figure 4F,H). For further confirmation, we applied the Rac1 inhibitor NSC 23766, which also interfered with long-distance transport, confirming the relevance of Rac1 activity in the regulation of the long-distance transport of EBOV NCLS (Figure 4H).

Taken together, these findings indicate that EBOV nucleocapsids exploit the canonical Rac1/WAVE1/Arp2/3 signaling pathway for long-distance transport of NCLS inside the cell. This signaling pathway classically induces lamellipodial protrusions, in which Rac1 acts coordinately with other upstream signals to activate actin regulators. Future studies shall reveal whether other actin-regulatory mechanisms are also involved in the viral life cycle, and how alterations in long-distance transport affect virus propagation.

## 4. Discussion

In this study, we have identified the Rac1/Wave1/Arp2/3 pathway as being involved in the actin-dependent transport of EBOV NCLS in human cell culture. Arp2/3 activity is also essential to inducing propulsive and polar-localized actin tails at the rear ends of NCLS, which can be robustly visualized in live cell imaging. Furthermore, the association of actin tails with EBOV NCLS and nucleocapsids can also be observed via super resolution in fixed samples.

Actin comet tails were initially characterized in *Listeria monocytogenes* in the late 1980s [40]. Since then, diverse types of intracellular pathogens have been identified as inducing actin polymerization at their surface. For instance, vaccinia virus protein A36 is able to recruit NCK and GRB2, which in turn recruit N-WASP to stimulate the Arp2/3 complex, or virus protein p78/83 mimics N-WASP and directly activates Arp2/3-induced actin polymerization [30,41]. Here, we showed that EBOV NCLS induce actin tails at one end, and actin tail formation is sensitive to the inhibition of Arp2/3 using CK666, indicating that the hijacking of the highly abundant and conserved Arp2/3 complex for induction of actin tails is a common mechanism in diverse types of pathogens without a common origin [30].

We further show that directed long-distance transport is regulated via Arp2/3 activity, yet the siRNA depletion of Arp3 in cells forming NCLS does not entirely block intracellular transport. Furthermore, the effects of siRNA treatment on track straightness appear more pronounced after Rac1 knockdown, when compared to the depletion of Arp3 or Wave1. These findings also suggest that other actin regulators downstream of Rac1 either compensate Arp2/3 activity, or synergistically regulate actin polymerization. One candidate could be the scaffolding protein IQGAP1 that interconnects multiple pathways of actin dynamics and interacts with Rac1. In cells infected with MARV, IQGAP1 was recruited to inclusion and to the read end of nucleocapsids, and the down-regulation of IQGAP1 resulted in the impaired release of MARV, suggesting a role for other major actin regulators in this process [14].

In addition, we also gained unprecedented evidence that Wave1 upstream of Arp2/3 is involved in regulating the long-distance transport of NCLS in Huh7 cells. Given that nucleocapsid transport in EBOV-infected cells does not depend on *N*-WASP [3], we concluded that other NFPs could be involved in the upstream regulation of Arp2/3. Further supporting this notion, N-WASP is typically involved in endocytotic events, where it regulates actin polymerization in a manner reminiscent of actin tails that propel pathogens through cells [42]. Consequently, enveloped viruses, such as vaccinia and EBOV, might profit from mimicking or recruiting N-WASP during their life cycle.

In recent years, additional NPFs, like WASH, WHAMM and JMY, have been described to promote actin tail formations at intracellular membranes like endosomes and autophagosomes [43,44,45]. In contrast, filoviral nucleocapsids travel in the cytosol without a membrane, and are highly structured protein complexes that probably utilize endocytosis-independent pathways for their transport, thereby likely avoiding membranous structures prior to their arrival at the plasma membrane. Wave1 and Rac1 signaling is considered primarily relevant to actin polymerization in lamellipodial cell protrusions [43], thus it is not surprising that long-distance NCLS transport is best observed in areas with a high activity of this pathway in Huh7 cells. Future studies using high resolution microscopy or electron microscopic approaches shall reveal whether and how actin regulators are actively recruited to EBOV nucleocapsids. It could be that at different stages of the nucleocapsid transport, from inclusion bodies to the budding sites, different actin tail-inducing machineries are exploited by the virus.

As described for other viruses, the long-distance transport of EBOV NCLS is accompanied by the induction of polar actin polymerization, likely resulting in the directionality of movement also observed in other actin tail-inducing pathogens and in in vitro reconstructions [41,46,47]. How this polar induction of actin polymerization is initiated is not understood in detail. One hypothesis derives from studies in *Listeria*, where it was shown that the surface protein ActA accumulates locally, likely during cell wall growth, which subsequently results in polar interactions with actin regulators [48]. In contrast, filoviral nucleocapsids are not enveloped when they leave inclusion bodies, and only encounter the viral proteins GP and VP40, which are transported independently, when they reach the plasma membrane to form infectious particles [49,50,51,52]. Thus, it remains unclear how this polar actin polymerization is induced in filoviral nucleocapsids, and which viral protein might be relevant for the actin polymerization. One explanation derives from a study investigating the structure of MARV capsids using cryo-electron microscopy, revealing that nucleocapsid assembly itself results in polar structures [10]. Here, it was demonstrated that MARV nucleocapsids are highly oriented towards the plasma membrane, with the pointed end of the nucleocapsid directed towards the plasma membrane prior to budding [11,53]. Thus, these findings support the notion that polar nucleocapsid assembly itself might result in specific protein conformations, thereby exposing the binding sites for cellular proteins that induce actin polymerization.

Taken together, our minimalistic transfection-based system reveals new opportunities to study the cellular transport of filoviral nucleocapsids, and to reliably quantify the dynamics of subviral structures. Future studies have to further characterize how filoviral proteins modulate highly spatial–temporal RhoGTPase signaling, and identify the direct interaction partners and structural changes that are required for long-distance transport during the EBOV life cycle.

## Supplementary Materials

The following are available online at https://www.mdpi.com/2073-4409/9/7/1728/s1, Figure S1: Analysis of tracks in cells treated with the Arp2/3 inhibitor CK666 in subgroups, Movie S1: NCLS induce actin tails during long-distance movement. Huh7 cells expressing EBOV proteins NP, VP35, VP24 and VP30-GFP (NCLS) and LifeAct-CLIP were monitored by time-lapse microscopy. Here, a single NCLS structure is followed showing a pulsative actin tail, Movie S2: NCLS movement and actin tails depend on Arp2/3 activity. A Huh7 cell expressing EBOV proteins NP, VP35, VP24 and VP30-GFP (NCLS) and LifeAct-CLIP was monitored by time lapse confocal microscopy. Note that NCLS capsids transported over long distances show actin tails (white arrow). The same cells were then incubated with the Arp2/3 inhibitor CK666 and reimaged. All long–distance movement is abolished while actin structures like filopodia and stress fibers remain unaffected.
