# Supplementary figures and images for: Ebola Virus Nucleocapsid-Like Structures Utilize Arp2/3 Signaling for Intracellular Long-Distance Transport
Source: Cells. 2020 Jul 19;9(7):1728. doi: 10.3390/cells9071728 (PMC7407605; doi:10.3390/cells9071728)

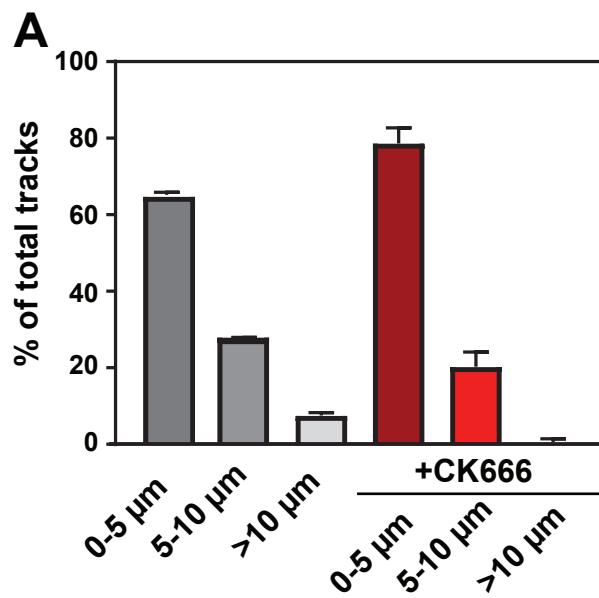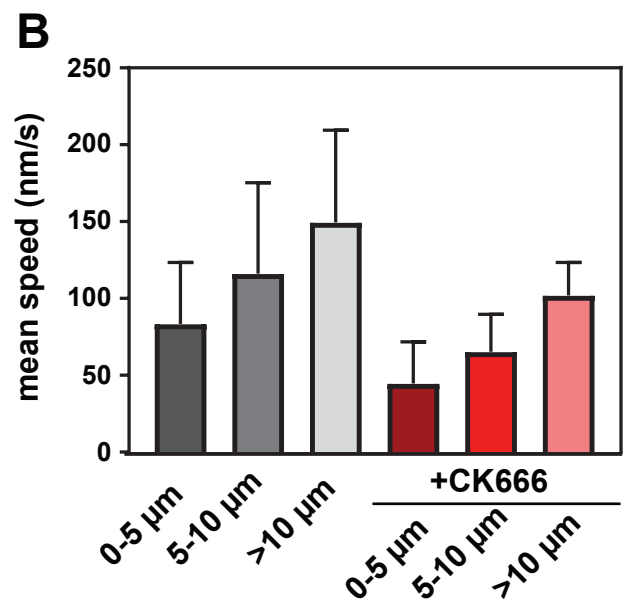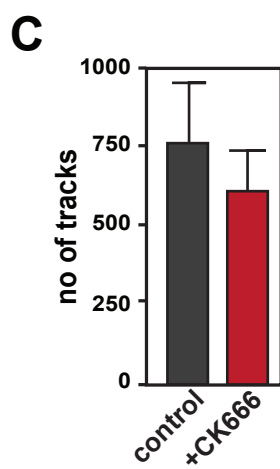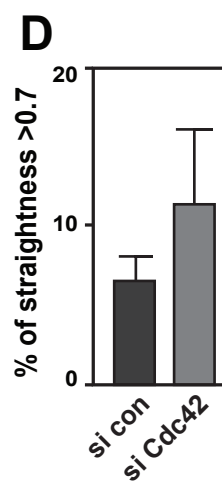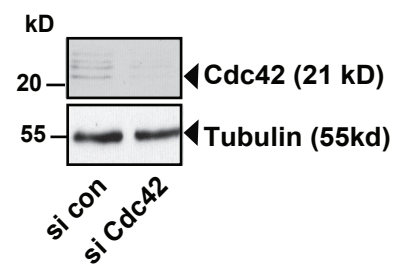

Supplement: Supplementary file 1 [file cells-09-01728-s001.zip › Supplement fig.pdf]
